# Intramuscular Contributions to Low-Frequency Force Potentiation Induced by a High-Frequency Conditioning Stimulation

**DOI:** 10.3389/fphys.2017.00712

**Published:** 2017-09-20

**Authors:** Arthur J. Cheng, Daria Neyroud, Bengt Kayser, Håkan Westerblad, Nicolas Place

**Affiliations:** ^1^Department of Physiology and Pharmacology, Karolinska Institutet Stockholm, Sweden; ^2^Faculty of Biology-Medicine, Institute of Sport Sciences, University of Lausanne Lausanne, Switzerland; ^3^Department of Physical Therapy, University of Florida Health Science Center Gainesville, FL, United States

**Keywords:** M-wave, intact single fiber, plantar flexors, muscle length, intracellular Ca^2+^

## Abstract

Electrically-evoked low-frequency (submaximal) force is increased immediately following high-frequency stimulation in human skeletal muscle. Although central mechanisms have been suggested to be the major cause of this low-frequency force potentiation, intramuscular factors might contribute. Thus, we hypothesized that two intramuscular Ca^2+^-dependent mechanisms can contribute to the low-frequency force potentiation: increased sarcoplasmic reticulum Ca^2+^ release and increased myofibrillar Ca^2+^ sensitivity. Experiments in humans were performed on the plantar flexor muscles at a shortened, intermediate, and long muscle length and electrically evoked contractile force and membrane excitability (i.e., M-wave amplitude) were recorded during a stimulation protocol. Low-frequency force potentiation was assessed by stimulating with a low-frequency tetanus (25 Hz, 2 s duration), followed by a high-frequency tetanus (100 Hz, 2 s duration), and finally followed by another low-frequency (25 Hz, 2 s duration) tetanus. Similar stimulation protocols were performed on intact mouse single fibers from *flexor digitorum brevis* muscle, whereby force and myoplasmic free [Ca^2+^] ([Ca^2+^]_i_) were assessed. Our data show a low-frequency force potentiation that was not muscle length-dependent in human muscle and it was not accompanied by any increase in M-wave amplitude. A length-independent low-frequency force potentiation could be replicated in mouse single fibers, supporting an intramuscular mechanism. We show that at physiological temperature (31°C) this low-frequency force potentiation in mouse fibers corresponded with an increase in sarcoplasmic reticulum (SR) Ca^2+^ release. When mimicking the slower contractile properties of human muscle by cooling mouse single fibers to 18°C, the low-frequency force potentiation was accompanied by minimally increased SR Ca^2+^ release and hence it could be explained by increased myofibrillar Ca^2+^ sensitivity. Finally, introducing a brief 200 ms pause between the high- and low-frequency tetanus in human and mouse muscle revealed that the low-frequency force potentiation is abolished, arguing that increased myofibrillar Ca^2+^ sensitivity is the main intramuscular mechanism underlying the low-frequency force potentiation in humans.

## Introduction

An enhancement of electrically-evoked low-frequency (~20–25 Hz) force immediately following a brief high-frequency tetanic stimulation has been readily observed in studies on human subjects (Collins et al., 2001, 2002; Baldwin et al., 2006; Klakowicz et al., 2006; Dean et al., 2007; Blouin et al., 2009; Lagerquist et al., 2009; Lagerquist and Collins, 2010; Bergquist et al., 2011a; Neyroud et al., 2016a). The potential functional implications of this phenomenon are not well-understood but an increased submaximal force for a given motoneuron input will improve the efficiency of muscle force generation. Previous studies have reported an almost doubling in low-frequency force immediately after a 2-s conditioning high-frequency stimulation (Collins et al., 2001, 2002; Baldwin et al., 2006; Lagerquist and Collins, 2010; Neyroud et al., 2016a) and attributed this phenomenon to central mechanisms and increased spinal motoneuron excitability (see Collins, 2007; Bergquist et al., 2011b for review). Support for a central mechanism comes from results showing high-frequency stimulation-induced asynchronous electromyographic (EMG) activity and H-reflex potentiation (Baldwin et al., 2006; Klakowicz et al., 2006; Bergquist et al., 2011a, 2012; Neyroud et al., 2016a). Moreover, some studies in humans show abolished low-frequency force potentiation when experiments were performed with complete anesthetic nerve blockade (Collins et al., 2001, 2002; Blouin et al., 2009; Lagerquist et al., 2009). However, there was no reported difference in low-frequency force potentiation before and after an anesthetic peripheral (afferent) nerve blockade in humans, as well as no change in low-frequency force potentiation in cats following sciatic nerve transection that abolishes central contributions (Frigon et al., 2011), which argues for intramuscular mechanisms for the force enhancement. Frigon et al. (2011) also observed a length dependency in the low-frequency force potentiation with larger effect at short muscle lengths, and they argued that this speaks in favor of an intramuscular mechanism.

Within skeletal muscle fibers, two Ca^2+^-dependent mechanisms might underlie the low-frequency force potentiation following high-frequency stimulation. First, during repeated high-frequency tetanic stimulation, rodent muscle fibers show an initial increase in the free myoplasmic [Ca^2+^] ([Ca^2+^]_i_) during repeated contractions (Westerblad and Allen, 1991; Lunde et al., 2001). This increase in tetanic [Ca^2+^]_i_ is typically accompanied by decreased high-frequency force during fatigue (Allen et al., 2008). However, low-frequency force might increase with the increase in [Ca^2+^]_i_ if minimal fatigue is induced. Second, an apparent increase in myofibrillar Ca^2+^ sensitivity might occur, because the increase in [Ca^2+^]_i_ during the high-frequency stimulation period will increase Ca^2+^ binding to troponin C and thereby more myosin heads can move tropomyosin from cross-bridge binding sites on the actin filament. Once tropomyosin has moved, the binding of additional cross bridges to neighboring sites will be facilitated and force might remain high for some time despite lowered [Ca^2+^]_i_ (Gordon et al., 2000; Abbate et al., 2002; Cheng et al., 2013; Bakker et al., 2017; Moss et al., 2017).

Here, we studied mechanisms underlying the low-frequency force potentiation following high-frequency stimulation. We hypothesized that the low-frequency force potentiation can involve intramuscular mechanisms and that these are muscle length- and Ca^2+^-dependent. These hypotheses were tested by performing experiments on human volunteers exposed to electrical stimulation of plantar flexor muscles and parallel mechanistic experiments on isolated mouse *flexor digitorum brevis* (FDB) fibers.

## Materials and methods

### Ethical approval

All human experiments were performed in agreement with the declaration of Helsinki and were approved by the Research Ethics Committee of the University Hospitals of Geneva (protocol 11-287). All participants gave their written informed consent before participation. All animal experiments complied with the Swedish Animal Welfare Act, the Swedish Welfare ordinance, and applicable regulations and recommendations from Swedish authorities. The study was approved by the Stockholm North Ethical Committee on Animal Experiments. A total of 11 mice (Janvier Laboratories, Le Genest-Saint-Isle, France) were used in these experiments. Mice were killed by rapid neck disarticulation, and whole FDB muscles removed from the hindlimbs.

### Human experiments

#### Subjects

Eleven healthy and physically active subjects (6 men and 5 women, 30 ± 7 years, 174 ± 7 cm, 71 ± 12 kg) volunteered to participate in this study after having been informed of the experimental procedures and possible risks. Before participation, each subject gave written informed consent. Six (3 men and 3 women, 31 ± 8 years, 172 ± 6 cm, 71 ± 12 kg) of these participants also took part in a second experimental part, described below.

#### Force recording

Subjects were seated with a knee angle of 140° and a trunk-thigh angle of 100° (180° = full extension). The knee angle was kept constant during the course of the experiment, carefully controlled with an electronic goniometer (SG150, Biometrics, Cwmfelinfach, United Kingdom). Extraneous movements of the upper body were limited by two crossover shoulder harnesses and a belt across the lower abdomen. Voluntary and electrically evoked forces developed by the plantar flexors (same muscle group as used in previous studies such as Bergquist et al., 2011a; Frigon et al., 2011; Neyroud et al., 2016a) were recorded using an isometric ergometer consisting of a custom-built chair equipped with a strain gauge (S2 1000N, sensitivity 2mV/V, HBM, Germany) fixed on a pedal. The ankle was strapped to the pedal at the ankle level as well as at the metatarsi level. In the first experimental session, the tests were performed at an intermediate (ankle angle of 90°), a shorter (105°), and a longer (75°) plantar flexor length in a random order, whereas in the second experimental session all tests involving a time delay during electrical stimulation were performed at intermediate muscle length (see Section Experimental Protocol). To limit the contribution of other muscle groups and to optimize force recordings, the upper leg was clamped down to the chair just proximal from the knee. Forces were recorded at 1 kHz using an analog-to-digital conversion system (MP150, BIOPAC, Goleta, USA).

#### Electromyographic (EMG) recording

EMG activity from the *soleus* muscle was recorded with pairs of silver chloride (Ag/AgCl) circular (recording diameter of 1 cm) surface electrodes (Kendall Meditrace 100, Tyco, Canada) positioned lengthwise over the middle of the muscle belly (according to SENIAM recommendations; Hermens et al., 2000) with an inter-electrode (center-to-center) distance of 2 cm. The reference electrode was placed on the patella of the ipsilateral leg. EMG signals were amplified (x1000) with a frequency window between 10 and 500 Hz, digitized at a sampling frequency of 2 kHz and recorded by the analog-to-digital conversion system.

#### Electrical stimulation

A high-voltage (maximal voltage 400 V) constant-current stimulator (model DS7AH, Hertfordshire, UK) was used to deliver electrical stimulation to the *triceps surae* muscle belly. Two 10 × 5 cm electrodes (Compex, Ecublens, Switzerland) were positioned over the *gastrocnemii* (~5 cm below the popliteal fossa) and *soleus* (~10 cm above the calcaneus) muscles. Pulse width was set to 1 ms for all stimulations.

#### Experimental protocol

Following a warm-up consisting of 8–10 submaximal contractions, maximal voluntary contraction (MVC) force was determined by asking the participants to produce a maximal contraction reaching maximal force within 2 s and to sustain a plateau for about 4–5 s. MVCs were separated by 1 min of rest and no more than 5% variation between the two last MVCs was tolerated.

The electrical stimulation intensity was then determined. The subjects were instructed to relax completely before each electrical stimulation. The current required to evoke a force of ~5–10% MVC using five pulses of 1-ms duration at 100 Hz was then determined (Frigon et al., 2011). This stimulation intensity was used for all subsequent stimulations and was re-determined when ankle angle was changed (24 ± 14, 17 ± 11, and 27 ± 14 mA at 75, 90, and 105° respectively). To assess the extent of low-frequency force potentiation, the plantar flexor muscles were electrically stimulated with a 25-Hz low-frequency tetanus for 2 s, followed by a 2-s 100-Hz tetanus (i.e., high frequency conditioning tetanus), and finally followed by another 25-Hz low-frequency tetanus of 2-s duration; this stimulation protocol is similar to that used in previous studies (Collins et al., 2001, 2002; Collins, 2007; Bergquist et al., 2011a, 2012; Frigon et al., 2011). This stimulation pattern was delivered two to three times at each of the three different ankle joint angles (75, 90, and 105°). The testing order of joint angles was counterbalanced across subjects. The stimulations were separated by ~1 min to minimize fatigue and to avoid contraction-dependent facilitation of the motor units (Gorassini et al., 2002a,b).

To further assess the mechanisms underlying low-frequency force potentiation, six of the participants took part in a second experimental session during which the stimulation protocol described above was performed except that a 200-ms pause was introduced between the high-frequency conditioning tetanus and the following low-frequency tetanus. These experiments were performed at an ankle angle of 90° (i.e., intermediate muscle length).

#### Data analysis

For all parameters, data from all trials were averaged when multiple trials were performed.

##### EMG

The peak to peak amplitude of 15 M waves were averaged between 1.25 and 1.85 s within each 25-Hz stimulation train (based on Lagerquist and Collins, 2010). Relative changes between the amplitudes measured before and after the 100-Hz conditioning stimulation were then calculated. H reflexes could only be observed in one subject and was therefore not measured for analysis.

##### Force

The force evoked during the last 1.5 s of each 25-Hz stimulation train was considered (Frigon et al., 2011). The magnitude of low-frequency force potentiation was calculated as the relative difference between 25-Hz forces delivered after (F_post_) vs. before (F_pre_) the 100-Hz conditioning tetanus, i.e., (F_post_ − F_pre_)/F_pre_.

### Animal experiments

#### Force and [Ca^2+^]_i_ measurements

Intact, single muscle fibers were mechanically dissected from FDB muscles (Cheng and Westerblad, 2017). The fiber was mounted in a chamber between an Akers 801 force transducer (Kronex technologies, Oakland, CA, USA) and an adjustable holder. The fiber length was adjusted to give maximum tetanic force (i.e., optimal length). Sarcomere length was measured as the average of 10 sarcomere lengths; experiments were performed at optimal length or with sarcomere length shortened or lengthened by 0.5 μm. The diameter of the fiber was measured at the optimal length and this measurement was used to calculate the cross-sectional area. The fiber was electrically stimulated with supramaximal current pulses (0.5-ms duration) given via platinum electrodes placed along the long axis of the fiber.

Fibers were superfused by a Tyrode solution containing (in mM): 121 NaCl, 5.0 KCl, 1.8 CaCl_2_, 0.5 MgCl_2_, 0.4 NaH_2_PO_4_, 24.0 NaHCO_3_, 0.1 EDTA, and 5.5 glucose. The solution was bubbled with 95% O_2_–5% CO_2_, giving a bath pH of 7.4. Foetal calf serum (0.2%) was added to the solution. Experiments were performed at ~31°C, unless otherwise stated, which is the *in vivo* physiological temperature for the mouse FDB muscle.

Fibers were microinjected with the fluorescent Ca^2+^ indicator indo-1 (Thermo Fisher Scientific, Stockholm, Sweden). The emitted fluorescence of indo-1 was measured with a system consisting of a Xenon lamp, a monochromator, and two photomultiplier tubes (Photon Technology International, Wedel, Germany). The excitation light was set to 360 nm, and the light emitted at 405 ± 5 and 495 ± 5 nm was measured by the photomultipliers. The ratio of the light emitted at 405 nm to that at 495 nm (R) was converted to [Ca^2+^]_i_ using the following equation:

$${\left[{\text{Ca}}^{2+}\right]}_{\text{i}}=K_{d}\beta (\text{R}-{\text{R}}_{\text{min}}){\left({\text{R}}_{\text{max}}-\text{R}\right)}^{-1}$$

The apparent dissociation constant of indo 1, *K*_*d*_, and the ratio of the 495 nm signals at very low and saturating [Ca^2+^]_i_, β, were obtained from Andrade et al. (1998) and amounted to 283 and 4.44 nM, respectively. R_min_ and R_max_ represent the ratios at very low and high [Ca^2+^]_i_, respectively, and were determined by repeated injections of 0.5 M EGTA or 1 M CaCl_2_ until stable ratios were obtained (Cheng and Westerblad, 2017). R_min_ was 0.65 and R_max_ was 5.6. Fluorescence and force signals were sampled online and stored on a computer for subsequent data analysis.

### Experimental protocol

We employed a stimulation protocol similar to that used in the human experiments: first 2 s of low-frequency stimulation, followed by 1 s of high-frequency (100 Hz) stimulation, and finally 2 s of low-frequency stimulation unless otherwise stated. In the mouse experiments, there was an ~2 s pause between the initial low-frequency stimulation and the following high-frequency stimulation due to a lag required to manually switch between stimulation frequencies. The second low-frequency stimulation started either immediately after the high-frequency stimulation or after a 200-ms pause. The low frequency ranged from 25 to 40 Hz and was for each fiber set so that 30–50% of the 100-Hz force was produced at optimal length in the unpotentiated state. In experiments with repeated tests, 10 min of rest was provided between stimulations. The order of testing was randomized in experiments performed at different sarcomere lengths. Force was measured as the peak during each stimulation period and [Ca^2+^]_i_ as the mean during each stimulation period. The magnitude of low-frequency force and [Ca^2+^]_i_ potentiation were calculated as the relative difference between measurements after vs. before the 100-Hz conditioning stimulation (i.e., same as for force potentiation in the human experiments).

### Statistics

Data are presented as mean ± SD. Data distribution was checked for normality (Shapiro-Wilk test) and depending on the outcome, parametric or non-parametric tests were applied. The comparison of MVC force between the three muscle lengths tested was performed with a one-way repeated measure ANOVA. Low frequency force potentiation and the associated M-wave amplitudes obtained in humans were tested with one sample *t*-tests at each length and the extent of potentiation was compared between the three lengths with a one-way repeated measure ANOVA. The comparison of force and tetanic [Ca^2+^]_i_ between the three muscle lengths tested in single fibers was performed with one-way repeated measures ANOVA. At each muscle length, statistically significant relative changes in force and tetanic [Ca^2+^]_i_ before and after the high-frequency conditioning tetanus in single fibers were assessed using paired *t*-tests. Comparisons of the extent of potentiation of force and tetanic [Ca^2+^]_i_ with and without a delay after the high-frequency conditioning stimulus was assessed using paired *t*-tests. Tukey's HSD *post-hoc* test was used when appropriate. The level of significance was set at *p* < 0.05. All statistical analyses were performed with Sigmaplot software for Windows (version 11; Systat, Chicago, USA).

## Results

### Electrically stimulated human muscle and isolated mouse muscle fibers show length-independent low-frequency force potentiation

**Figure 1:**
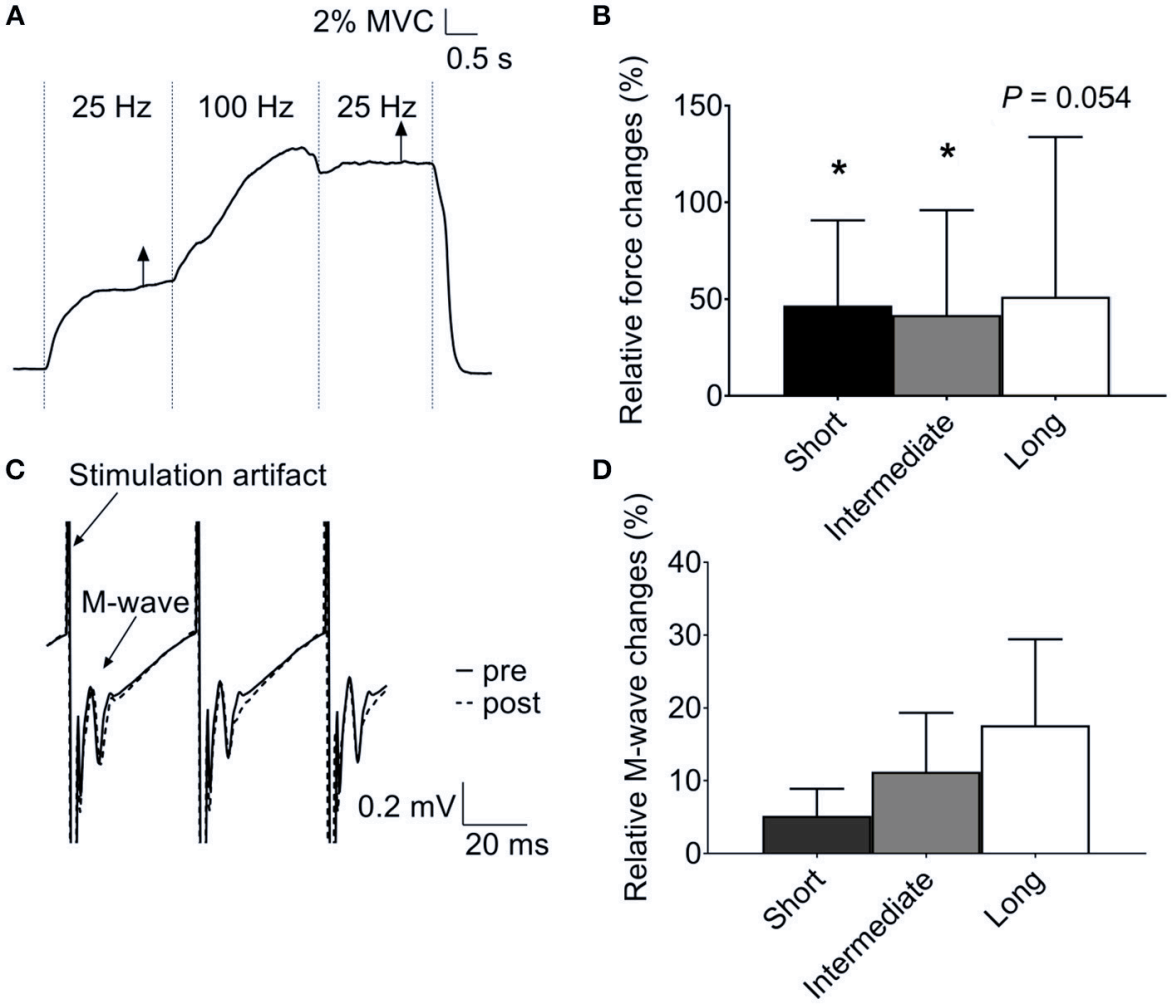
Increased low-frequency force potentiation after a high-frequency conditioning tetanus in human plantar flexors. **(A)** Original records of the force signal illustrating the low-frequency force potentiation at an ankle angle of 90°. **(B)** Mean data (± SD) (*n* = 11) of the relative low-frequency force potentiation induced by the conditioning 100-Hz tetanus at short, intermediate, and long muscle length (i.e., ankle angle of 105, 90, and 75°, respectively) showed no length-dependency in force potentiation. **(C)** Original traces of *soleus* electromyographic activity from the same subject as in **(A)**, recorded at the time indicated by the vertical arrows in **(A)**. Note that no obvious difference was observed between the M wave following each stimulation artifact before (full line) and after (dashed line) the 100-Hz stimulation. **(D)** Mean data (±SD) (*n* = 8) of relative changes in *soleus* M-wave amplitude after the conditioning 100-Hz stimulation at the three muscle lengths showed no length-dependent difference in membrane excitability. ^*^Significantly increased relative to the initial low-frequency tetanus, *p* < 0.05.

MVC force in human plantar flexors was lower (*p* < 0.05, *n* = 11) at the shortest length (387 ± 182 N), with no difference between the intermediate (670 ± 341 N) and long (831 ± 458 N) muscle length. The original force record from an electrically evoked contraction at the intermediate length (90° ankle angle) in Figure 1A shows a marked force potentiation at 25 Hz after the 100-Hz stimulation period. A force potentiation (~30%) during the second 25-Hz stimulation was observed at all three muscle lengths, although it did not reach statistical significance at the long length (*p* = 0.054) (Figure 1B). Contrary to our hypothesis, the magnitude of this force potentiation was similar (*p* > 0.05) at the three lengths (Figure 1B).

Unlike force, *soleus* M-wave amplitude was not significantly affected by the 100-Hz conditioning stimulation at any of the muscle lengths (*p* > 0.05) (Figures 1C,D).

Intact single FDB fibers studied at three different lengths showed a main effect of force across lengths with the greatest 100-Hz force (336 ± 39 kN m^−2^; *n* = 8) at the optimal (i.e., intermediate) sarcomere length (2.8 ± 0.04 μm), and about 20% lower force at longer (3.3 ± 0.1 μm) and shorter (2.3 ± 0.1 μm) sarcomere length. Despite the greater force at optimal length, [Ca^2+^]_i_ during 100-Hz stimulation did not show any length dependency (*p* > 0.05), being 1.5 ± 0.5 μM at optimal length, 1.7 ± 1.0 μM at long length, and 1.3 ± 0.2 μM at short length.

**Figure 2:**
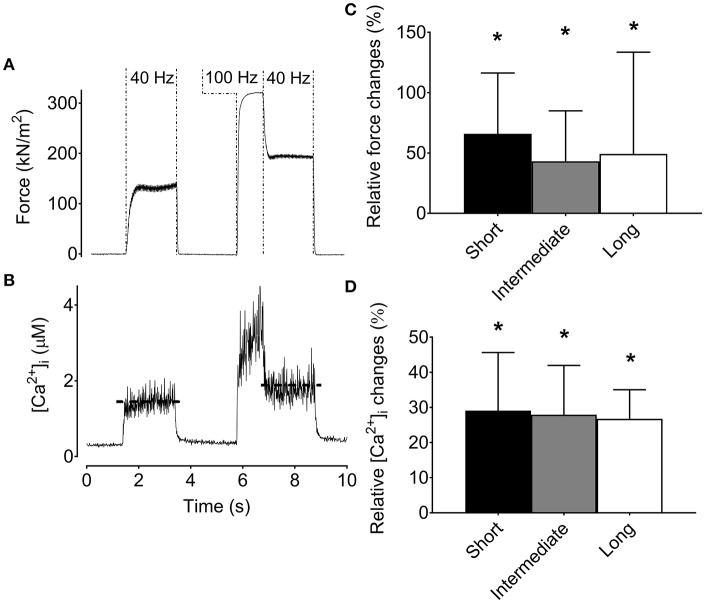
The increase in low-frequency force and [Ca^2+^]_i_ after a high-frequency conditioning tetanus is not muscle length-dependent in mouse *flexor digitorum brevis* intact single fibers. Original recordings of the force **(A)** and [Ca^2+^]_i_ **(B)** during stimulation in a mouse *flexor digitorum brevis* single fiber, and mean (±SD) values (*n* = 8) of the relative increase in force **(C)** and [Ca^2+^]_i_ **(D)** for the low-frequency tetanus following the high-frequency conditioning tetanus. The horizontal dashed lines in the [Ca^2+^]_i_ recording reflect the mean [Ca^2+^]_i_. ^*^Significantly increased relative to the initial low-frequency tetanus at each length, *p* < 0.05.

The typical force and [Ca^2+^]_i_ records in Figures 2A,B were obtained at optimal length and show marked increases in both low-frequency force and [Ca^2+^]_i_ after the high-frequency stimulation. Average data show increases in low-frequency force and [Ca^2+^]_i_ after the high-frequency stimulation at all lengths and there was no statistically significant length dependency (*p* > 0.05) (Figures 2C,D).

### The low-frequency force potentiation in human muscle is abolished by a 200-ms pause

**Figure 3:**
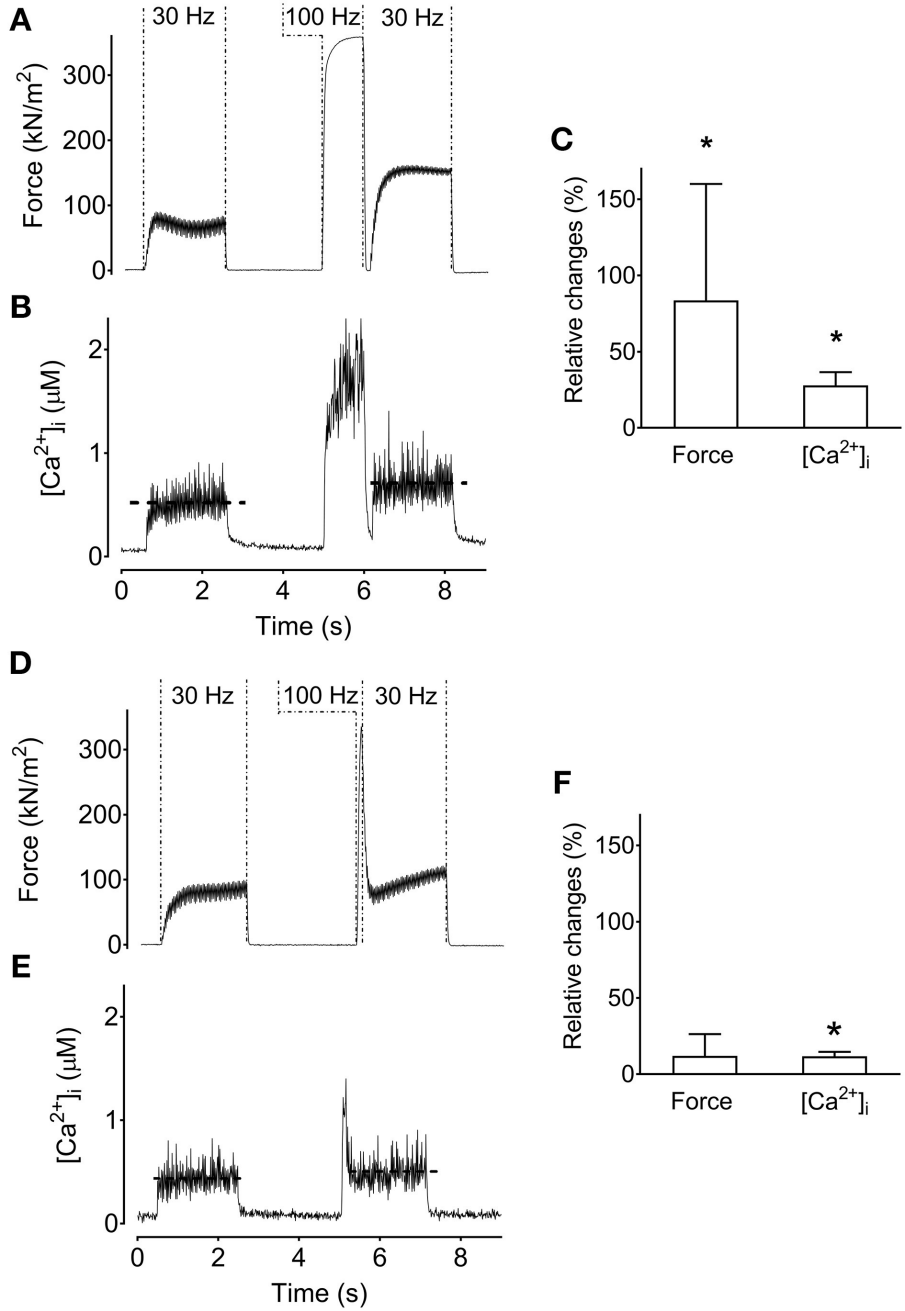
Increased tetanic [Ca^2+^]_i_ can increase low-frequency force. Original recordings of the force **(A)** and [Ca^2+^]_i_ **(B)** from a mouse *flexor digitorum brevis* single fiber during stimulation which introduced a 200-ms pause between the high-frequency conditioning tetanus and the following low-frequency tetanus. Mean (±SD) values of the relative change in low-frequency force and tetanic [Ca^2+^]_i_ (*n* = 10) show that the increased force corresponds to increased tetanic [Ca^2+^]_i_ **(C)**. Original recordings of the force **(D)** and [Ca^2+^]_i_ **(E)** under a stimulation paradigm that minimally increased tetanic [Ca^2+^]_i_ by evoking a brief 100-ms 100-Hz tetanus. **(F)** Mean (±SD) values of the relative change in low-frequency force and tetanic [Ca^2+^]_i_ (*n* = 3) show that force is not significantly increased when the increase in tetanic [Ca^2+^]_i_ is minimal. The horizontal dashed lines in the [Ca^2+^]_i_ recording reflect the mean [Ca^2+^]_i_. ^*^Significantly increased relative to the initial low-frequency tetanus, *p* < 0.05.

The results from single FDB fibers described above show that the low-frequency force potentiation can have an intramuscular origin. To reveal the mechanism(s) involved, we performed additional experiments on FDB fibers where a 200-ms pause was introduced after the high-frequency stimulation (all these experiments were performed at optimal length). The rationale behind these experiments is that the pause would diminish an apparent increase in myofibrillar Ca^2+^ sensitivity due to more attached cross-bridges immediately following the high-frequency stimulation (Gordon et al., 2000; Abbate et al., 2002). On the other hand, the pause would not affect a force potentiation caused by increased [Ca^2+^]_i_. The original records and average data in Figures 3A–C show increased low-frequency force and [Ca^2+^]_i_ after the high-frequency stimulation period even when the stimulation was interrupted by a 200-ms pause (*p* < 0.05). Thus, with this stimulation protocol, the low-frequency force potentiation would be due to the higher [Ca^2+^]_i_ after the high-frequency stimulation. Next we searched for a protocol where low-frequency [Ca^2+^]_i_ was minimally potentiated after the high-frequency stimulation. This was achieved by decreasing the conditioning 100-Hz stimulation period to a brief 100-ms duration. Figures 3D–F show that this stimulation protocol did not result in any significant increase in low-frequency force following the high-frequency conditioning tetanus. Thus, the increase in [Ca^2+^]_i_ induced by the longer 1-s high-frequency tetanus can explain the low-frequency force potentiation in Figures 3A–C.

**Figure 4:**
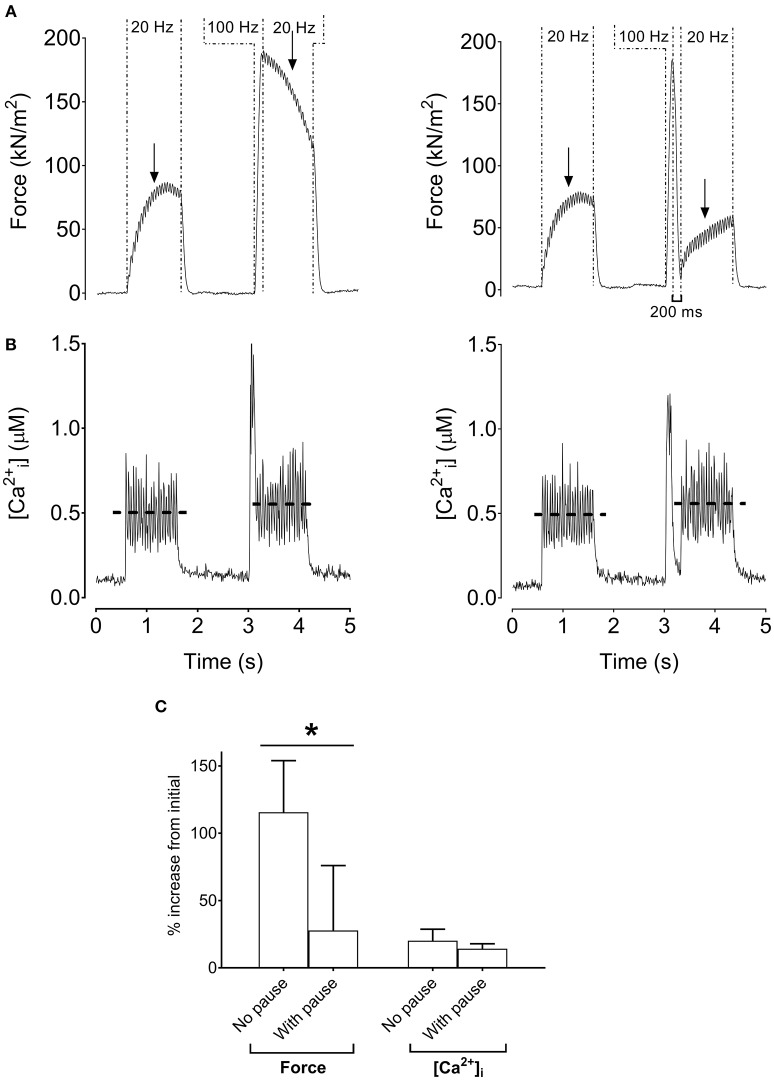
Increased low-frequency force without increased tetanic [Ca^2+^]_i_ in mouse single fibers. Original recordings of force and [Ca^2+^]_i_ in mouse *flexor digitorum brevis* intact single fibers cooled to 18°C during the stimulation sequence (1 s at 20 Hz, 100 ms at 100 Hz, and 1 s at 20 Hz) without **(A)** or with **(B)** a 200-ms pause following the 100-Hz tetanus. Mean (±SD) values showing relative increases of force and tetanic [Ca^2+^]_i_ (*n* = 4) with and without a 200-ms pause **(C)**. The horizontal dashed lines in the [Ca^2+^]_i_ recording reflect the mean [Ca^2+^]_i_. Arrows indicate the 500-ms time point from the start of the 20-Hz tetanus where force was measured. ^*^Significantly different force between stimulations without and with a pause after the 100-Hz tetanus, *p* < 0.05.

Previously we noted the much slower contractile properties of human vs. rodent muscle, and a better match can be achieved by reducing the temperature of rodent muscle to 18°C (Neyroud et al., 2016b). Figure 4 shows the stimulation protocol with a 100-ms high-frequency conditioning tetanus (i.e., which minimally increased [Ca^2+^]_i_) with and without a 200-ms pause between the high-frequency tetanus and the following low-frequency tetanus. Interestingly, the low-frequency force potentiation was markedly greater after the stimulation protocol without than with a 200-ms pause (*p* < 0.05). Thus, with this stimulation protocol, the low-frequency force potentiation would be due to an apparent increase in myofibrillar Ca^2+^ sensitivity.

**Figure 5:**
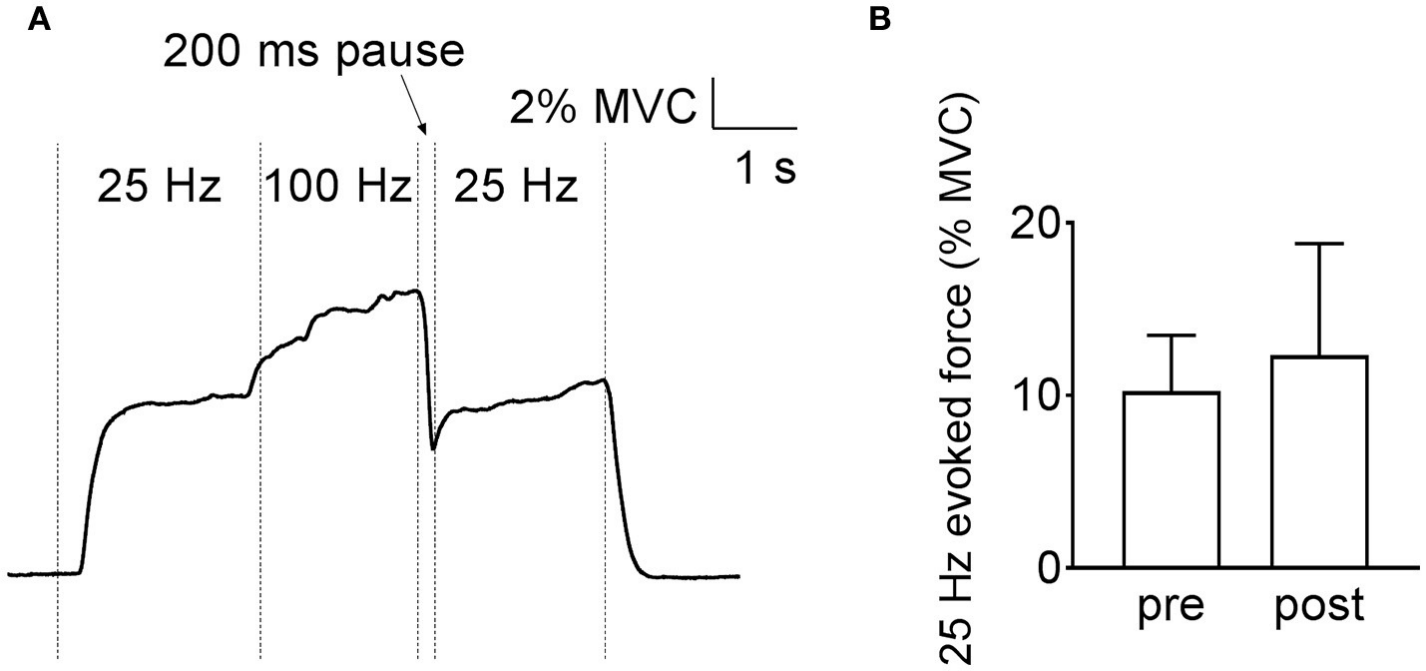
Low-frequency force potentiation is abolished by adding a 200-ms pause after the high-frequency conditioning stimulation in human plantar flexors. Original recording of the force **(A)** and mean (±SD) values (*n* = 6) **(B)** show that the low-frequency force is not different before and after the high-frequency stimulation.

A final set of experiments was performed to distinguish between the two tentative Ca^2+^-dependent intramuscular mechanisms of the low-frequency force potentiation in human plantar flexor muscles (see Figure 1). For this purpose, plantar flexor muscles of six of the participants were stimulated at the intermediate muscle length and a 200-ms pause was introduced after the conditioning 100-Hz stimulation. Figure 5 shows similarly sized low-frequency force before and after the high-frequency stimulation, i.e., the introduction of the pause abolished the low-frequency force potentiation. Thus, the force potentiation in the human muscles would be due to an apparent increase in myofibrillar Ca^2+^ sensitivity.

## Discussion

In the current study, we investigated whether intramuscular mechanisms might contribute to the low-frequency force potentiation observed after a brief conditioning high-frequency (100 Hz) stimulation. In agreement with our hypothesis, the results showed that direct electrical stimulation of human muscles *in vivo* and also in isolated mouse muscle fibers resulted in low-frequency force potentiation. We also hypothesized that the low-frequency force potentiation would depend on the muscle length, but the results showed no length-dependency either in human muscles or mouse single fibers. Finally, we hypothesized that low-frequency force potentiation involves a Ca^2+^-dependent mechanism. The mouse single muscle fiber experiments revealed two Ca^2+^-dependent mechanisms: increased SR Ca^2+^ release and increased myofibrillar Ca^2+^ sensitivity. Experiments with and without a 200-ms pause between the high-frequency stimulation and the subsequent low-frequency stimulation revealed increased myofibrillar Ca^2+^ sensitivity as the main intramuscular mechanism underlying the low-frequency force potentiation in human muscle.

A length-dependency with larger low-frequency force potentiation at shorter lengths was previously used to argue for an intramuscular mechanism (Frigon et al., 2011). Conversely, no length dependency was found in either human or mouse muscles in the present study. In our human experiments, we adjusted the electrical stimulation intensity to achieve a similar relative force at all muscle lengths, which meant larger currents at short and long lengths. Frigon et al. (2011), on the other hand, used the same electrical stimulation intensity for all muscle lengths. Thus, in their experiments the initial low-frequency force is likely to be lower at short lengths, hence leaving more room for a low-frequency force potentiation after the high-frequency burst. Furthermore, while the shortest plantar flexor length tested in the present study was sufficiently shortened to show a 50% lower MVC force than at the longest length, we cannot rule out that a length-dependency of low-frequency force potentiation may still exist at even shorter muscle lengths as previously shown by Frigon et al. (2011). Nevertheless, an intramuscular effect is still supported by Frigon et al. (2011) who showed that the extent of low-frequency force potentiation was unaffected by an afferent nerve block. This is in accordance with our finding that low-frequency force potentiation can be induced in isolated mouse muscle fibers.

Both increased Ca^2+^ release and/or increased myofibrillar Ca^2+^ sensitivity might potentiate muscle force (Allen et al., 2008). To disentangle the role played by these two potential mechanisms, a 200-ms pause was introduced between the 100-Hz burst and the subsequent 25-Hz stimulation bout. The rationale behind this experiment was that low-frequency force potentiation caused by increased [Ca^2+^]_i_ should not be diminished by a brief pause. This was further supported by experiments where a brief 100-ms high-frequency conditioning tetanus was used and where [Ca^2+^]_i_ was minimally increased, leading to no apparent force potentiation (see Figures 3D–F).

We previously noted that contractile kinetics are markedly faster in rodent than human muscle and they are better matched by cooling the isolated rodent muscle to 18°C (Neyroud et al., 2016b). Intriguingly, the brief 100-ms high-frequency conditioning tetanus led to increased force at 18°C despite [Ca^2+^]_i_ not remaining markedly elevated. Indeed, summation of submaximal force is improved by decreasing muscle temperature, which slows force relaxation (Ranatunga, 1980; Davies et al., 1982; Ranatunga et al., 1987; de Ruiter et al., 1999). On the other hand, low-frequency force potentiation disappeared following the pause introduced after the high-frequency conditioning tetanus. These findings point to an apparent increase in myofibrillar Ca^2+^ sensitivity as the most likely mechanism for low-frequency force potentiation. The difference in low-frequency force potentiation with or without a pause in the current study is unlikely explained by myosin light-chain phosphorylation because dissociation of Ca^2+^-calmodulin from myosin light-chain kinase upon muscle relaxation has a long de-activation half time of ~1.3 s and hence would not be diminished during a brief 200-ms pause (Sweeney et al., 1993).

Previously, a brief increase in [Ca^2+^]_i_ due to two- to three-pulse high-frequency (150–200 Hz) stimulation was sufficient to potentiate low-frequency force (Abbate et al., 2002; Cheng et al., 2013; Bakker et al., 2017). A proposed mechanism is that this low-frequency force potentiation is due to a “crossbridge-induced increase in myofibrillar Ca^2+^ sensitivity” (Cheng et al., 2013; Moss et al., 2017). Here, already attached crossbridges activated by the brief high [Ca^2+^]_i_ may facilitate binding of crossbridges to neighboring binding sites despite the lowered [Ca^2+^]_i_ during the subsequent low-frequency stimulation (Gordon et al., 2000; Fitzsimons and Moss, 2007) This mechanism would explain why a 200-ms pause, which disengages crossbridges during a brief relaxation, can abolish the low-frequency force potentiation. Although our mouse fiber experiments showed that both increased Ca^2+^ release and increased myofibrillar Ca^2+^ sensitivity can induce the low-frequency force potentiation, the disappearance of the low-frequency force potentiation with a pause in the human experiments suggests increased myofibrillar Ca^2+^ sensitivity as the predominant intramuscular mechanism.

Strengths of the mouse fiber experiments are that changes in SR Ca^2+^ release and myofibrillar Ca^2+^ sensitivity can be assessed in real-time in intact living cells, whereas only recently have these techniques been applied on isolated intact human single fibers (Olsson et al., 2015; Cheng and Westerblad, 2017). A focus of the current experiments was to use findings from mouse single fibers to reveal potential intramuscular processes involved in low-frequency force potentiation in humans. However, limitations in the translation between results from mouse single fibers and intact human muscles must be acknowledged and future studies indeed remain to get a detailed understanding of processes operating in human skeletal muscle. In addition, spinal (neuronal) mechanisms may also be involved in the low-frequency force potentiation in an intact neuromuscular system (Collins et al., 2001, 2002; Baldwin et al., 2006; Klakowicz et al., 2006; Blouin et al., 2009; Lagerquist et al., 2009; Bergquist et al., 2011a, 2012; Neyroud et al., 2016a). It is evident that future investigations are needed to delineate the contribution of central vs. peripheral mechanisms to low-frequency force potentiation.

In conclusion, the present study shows that intramuscular factors can contribute to the low-frequency force potentiation, and increased myofibrillar Ca^2+^ sensitivity is the major intramuscular factor residing in humans. The potential functional implication of low-frequency force potentiation is that submaximal force is more easily sustained following a brief maximal contraction, which increases the efficiency of force generation for a given neural input. Myofibrillar Ca^2+^ sensitivity acutely declines during fatiguing exercise (Allen et al., 2008), and it is chronically decreased in certain diseases (Friedrich et al., 2015; Yamada et al., 2015; Joureau et al., 2017) and in aging (Lamboley et al., 2015). Thus, the increased myofibrillar Ca^2+^ sensitivity observed in the present study can be an important mechanism to counteract decreases in submaximal force in healthy and diseased states.

## Author contributions

AC and DN contributed equally to this work. AC, DN, BK, HW, and NP contributed to the conception and design of the study, participated in the analysis and interpretation of the data. AC, DN, and NP were responsible for data collection. All authors were involved in writing the manuscript and approved the final version. All authors agreed to be accountable for all aspects of the work in ensuring that questions related to the accuracy or integrity of any part of the work are appropriately investigated and resolved. All persons designated as authors qualify for authorship, and all those who qualify for authorship are listed. Human experiments were performed in the Institute of Movement Sciences and Sports Medicine of Geneva University, Switzerland and in the Institute of Sport Sciences of the University of Lausanne, Switzerland. All animal experiments were performed at the Cellular Muscle Function Laboratory in the Department of Physiology and Pharmacology, Karolinska Institutet, Stockholm, Sweden.

### Conflict of interest statement

The authors declare that the research was conducted in the absence of any commercial or financial relationships that could be construed as a potential conflict of interest.
